# Low- versus high-power holmium: YAG laser strategies affect operative efficiency in suction-assisted mini-PCNL in a randomized controlled trial

**DOI:** 10.1038/s41598-026-52978-7

**Published:** 2026-05-11

**Authors:** Thanakorn Sirajarus, Kun Sirisopana, Surawach Piyawannarat, Yada Phengsalae, Premsant Sangkum, Wisoot Kongchareonsombat, Chinnakhet Ketsuwan

**Affiliations:** Division of Urology, Department of Surgery, Faculty of Medicine Ramathibodi Hospital, Mahidol University, 270 Rama VI Road, Thung Phayathai, Ratchathewi, Bangkok, 10400 Thailand

**Keywords:** Mini-PCNL, Holmium:YAG laser, Laser strategies, Suction-assisted PCNL, Renal calculi, Diseases, Medical research, Urology

## Abstract

To compare operative efficiency and perioperative outcomes between low-power (LP) and high-power (HP) holmium: yttrium aluminum–garnet (Ho: YAG) laser strategies in suction-assisted mini–percutaneous nephrolithotomy (mini-PCNL), we conducted a prospective randomized controlled trial including 100 adult patients with renal calculi randomized 1:1 to LP (*n* = 50) or HP (*n* = 50) settings. All procedures were performed using suction-assisted mini-PCNL. The primary outcome was total operative time, with secondary outcomes including laser activation time, total laser energy, energy density, ablation speed, stone-free rate (SFR) at 6 weeks, and complications. Baseline characteristics were comparable between groups. The HP group demonstrated significantly shorter operative time (90.7 ± 18.7 vs. 112.1 ± 40.3 min; *P* = 0.001) and laser activation time (1893 ± 499 vs. 2548 ± 1001 s; *P* = 0.001). Although total energy delivery was higher in the HP group (37.6 vs. 25.1 kJ; *P* = 0.001), ablation speed was significantly greater (1.6 vs. 1.2 mm³/s; *P* = 0.002), while the LP group showed greater energy efficiency (10.9 vs. 16.4 J/mm³; *P* = 0.002). Six-week SFR (86.0% vs. 88.0%; *P* = 0.766), complication rates, and hospital stay were comparable. Within a suction-assisted mini-PCNL setting, HP Ho: YAG laser strategies were associated with improved operative efficiency, primarily reflected by shorter operative and lasing times, at the expense of lower energy efficiency, without differences in short-term clinical outcomes. These findings should be interpreted as reflecting differences in clinically applied laser strategies rather than isolated laser power effects.

**Supplementary Information:**

The online version contains supplementary material available at 10.1038/s41598-026-52978-7.

## Introduction

Percutaneous nephrolithotomy (PCNL) remains the gold standard for large renal calculi (> 20 mm)^1,2^. However, the morbidity associated with conventional large-tract PCNL has accelerated the shift toward miniaturized techniques^3–5^. Mini-percutaneous nephrolithotomy (mini-PCNL, tract size ≤ 22 Fr) reduces bleeding and parenchymal injury but may be associated with reduced efficiency of stone clearance compared to standard PCNL^6,7^. The integration of suction-assisted access sheaths has partially addressed this limitation by facilitating active fragment evacuation and improving operative efficiency^8^.

Laser lithotripsy is the cornerstone of stone disintegration in mini-PCNL. Conventional low-power (LP) holmium: yttrium aluminum–garnet (Ho: YAG) laser settings are effective for fragmentation, while modern systems enable higher-frequency pulse modulation and alternative lithotripsy strategies that may accelerate stone disintegration^9–11^. However, the clinical impact of these different laser strategies on operative efficiency remains an area of ongoing investigation. Importantly, in contemporary practice, these strategies often involve overlapping power outputs and should be interpreted as combinations of pulse energy and frequency rather than strictly distinct power levels.

Few randomized trials have directly compared low- versus high-power laser strategies within the context of suction-assisted mini-PCNL^12,13^. Therefore, this study aimed to evaluate whether clinically applied high-power Ho: YAG laser strategies improve operative efficiency compared to low-power strategies, while also assessing perioperative outcomes including stone-free rate and complications.

## Patients and methods

### Patients

This prospective, randomized controlled trial was conducted at the Faculty of Medicine, Ramathibodi Hospital, Mahidol University, Thailand, between April 2023 and February 2024. All methods were performed in accordance with the relevant guidelines and regulations, including the Declaration of Helsinki and the Consolidated Standards of Reporting Trials (CONSORT) 2010 guidelines for reporting randomized controlled trials. Ethical approval was obtained from the Institutional Review Board of the Faculty of Medicine at Ramathibodi Hospital (COA. MURA2023/230), and the trial was registered with the Thai Clinical Trials Registry (TCTR20230420001) on April 20, 2023. Written informed consent was obtained from all participants prior to enrollment.

Adult patients (≥ 18 years) with renal calculi scheduled for mini-PCNL were screened for eligibility. Exclusion criteria included general contraindications to surgery, pregnancy, skeletal deformities precluding proper positioning, uncorrectable coagulopathy, pyonephrosis, active upper urinary tract infection without adequate antibiotic control, a positive preoperative urine culture, and an inability to complete postoperative imaging follow-up.

### Randomization and masking

Eligible patients were randomly assigned in a 1:1 ratio to either the LP or HP holmium: yttrium aluminum–garnet (Ho: YAG) laser lithotripsy group using a computer-generated randomization sequence kept in sequentially numbered, opaque sealed envelopes. The patients and postoperative outcome assessors were blinded to the treatment allocation. Blinding the operating surgeon was not feasible because of the distinct audible differences in laser frequency. However, the operative time was recorded by an independent circulating nurse to minimize potential performance bias.

### Surgical technique and perioperative management

All procedures were performed using a standardized operative protocol by a single experienced surgeon. The patients were positioned in the Galdakao-modified supine Valdivia position. Percutaneous renal access was obtained under fluoroscopic guidance. In all cases, a 16 Fr ClearPetra disposable nephrostomy sheath (Well Lead Medical Co., Ltd., Guangzhou, China) was utilized to facilitate active fragment evacuation and to maintain low intrarenal pressure. Suction was connected to a wall suction unit maintained at continuous negative pressure. However, the level of suction was dynamically adjusted intraoperatively according to visibility and fragment clearance, reflecting routine clinical practice. Stone fragments were primarily evacuated using continuous suction, and auxiliary extraction devices such as baskets or Perc-N-Circle were not routinely required. This approach was applied consistently across both study groups.

Laser lithotripsy was performed using a 120-W Ho: YAG laser (Lumenis, San Jose, CA, USA) with a 550 μm core laser fiber. Irrigation was maintained using the UROMAT E.A.S.I. pressure-controlled double-roller pump (Karl Storz, Tuttlingen, Germany), with irrigation pressure titrated to maintain clear visualization.

This study was designed to compare clinically applied LP and HP laser strategies rather than isolated parameters, reflecting real-world practice where pulse energy and frequency are dynamically adjusted. Due to these combinations, partial overlap in power output between groups was present. The corresponding power output (calculated as pulse energy × frequency) ranged from approximately 10–30 W in the LP group and 12–40 W in the HP group, reflecting partial overlap between the two strategies. Therefore, the comparison should be interpreted as a comparison of clinically applied laser strategies rather than strictly distinct power levels.

In the LP group, the settings included fragmentation (1.0 J, 10 Hz, short pulse), dusting (0.5–0.8 J, 15–20 Hz, long pulse), and pop-corning (1.2–1.5 J, 15–20 Hz, short pulse). Pop-corning is a low-frequency, higher-energy lithotripsy technique used to fragment residual stone pieces within a confined space, often resulting in larger fragments and increased retropulsion.

In the HP group, the settings included fragmentation (2.0 J, 15 Hz, short pulse), dusting (0.2–0.3 J, 60–80 Hz, long pulse), and pop-dusting (0.5 J, 50–80 Hz, short pulse). Pop-dusting refers to a high-frequency, low-energy technique that promotes continuous fine fragmentation into dust-like particles with reduced retropulsion.

Laser settings were titrated intraoperatively according to stone hardness but remained within the assigned laser strategy group. At the end of the procedure, a 6-Fr double-J ureteral stent was routinely placed.

### Data collection and outcome measures

Baseline demographic and stone-related variables were recorded preoperatively, including age, sex, body mass index, comorbidities, and the American Society of Anesthesiologists (ASA) physical status classification (Class I–VI)^14^. Stone size was defined as the maximum diameter of the largest stone measured on preoperative imaging. The number of stones per patient was also recorded and categorized as 1, 2, 3, or ≥ 4. Intraoperative and postoperative data were prospectively collected, including total operative time (defined as time from cystoscopy insertion to ureteral stent placement), laser activation time, total laser energy delivered, hemoglobin and hematocrit levels, estimated blood loss, transfusion requirement, length of hospital stay, and perioperative complications. Complications were graded according to the Clavien–Dindo classification^15^.

Stone-free status was assessed at 6 weeks postoperatively using noncontrast computed tomography of the kidney, ureter, and bladder (CT KUB). Stone-free status was defined as the absence of residual stones or the presence of clinically insignificant residual fragments (CIRF) ≤ 4 mm. Zero-fragment status was defined as complete absence of residual fragments on postoperative CT imaging. All postoperative assessments were performed by an independent investigator blinded to the treatment allocation.

### Laser efficiency parameters

Laser lithotripsy efficiency was evaluated using laser activation time (seconds), total laser energy delivered (joules), stone ablation speed (mm³/s), and energy required to ablate 1 mm³ of stone (J/mm³). Laser activation time was defined as the cumulative laser firing duration recorded by the laser console.

Stone volume was calculated from preoperative CT imaging using the validated ellipsoid formula (stone volume = length × width × depth × π/6). Stone ablation speed was calculated as stone volume divided by laser activation time, and energy efficiency was calculated as total laser energy divided by stone volume. These laser efficiency parameters were considered exploratory secondary analyses.

Preoperative stone volume was used as a surrogate denominator for all efficiency calculations, regardless of postoperative residual fragment status. Residual fragments were not directly incorporated into these calculations.

### Outcomes

The primary outcome was the total operative time. Secondary outcomes included laser activation time, total laser energy delivered, stone ablation speed, energy per unit stone volume, stone-free rate at 6 weeks, estimated blood loss, hemoglobin drop, perioperative complications, and length of hospital stay.

### Sample size calculation

Sample size estimation was based on the primary endpoint of the total operative time. Using data from a previously published mini-PCNL study^12^ reporting a mean difference of approximately 24 min between LP and HP Ho: YAG laser settings with a standard deviation of 15–17 min, a conservative clinically meaningful difference of 10 min was prespecified. Based on these assumptions, 40 renal units per group were required to achieve 80% statistical power with a two-sided α level of 0.05. To account for potential dropouts or incomplete follow-up, the target sample size was increased to 50 renal units per group.

### Statistical analysis

Statistical analyses were performed using Stata version 14.2 (StataCorp, College Station, TX, USA). Analyses were conducted based on the intention-to-treat principle. Continuous variables were assessed for normality using the Shapiro–Wilk test and visual inspection of histograms. Normally distributed data are presented as mean ± standard deviation and were compared using the independent-samples t-test. Non-normally distributed data are presented as median (interquartile range) and were compared using the Mann–Whitney U test. Categorical variables are presented as frequencies and percentages and were compared using the chi-square test or Fisher’s exact test, as appropriate. A two-sided P-value < 0.05 was considered statistically significant.

Continuous variables are presented as mean ± standard deviation in the main tables, and corresponding median (interquartile range) values are additionally provided in Supplementary Table S1 to allow assessment of data distribution.

## Results

**Fig. 1:**
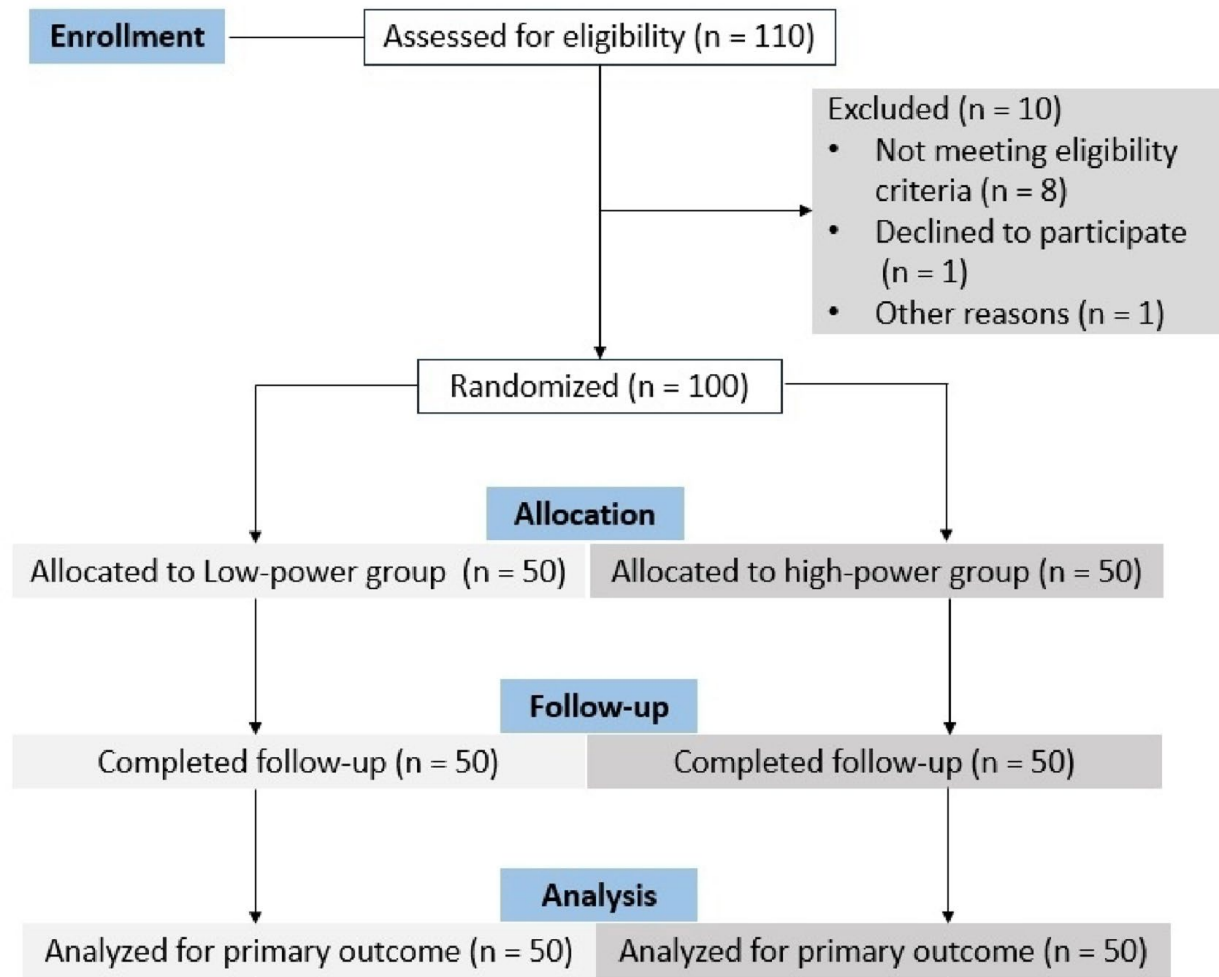
Flow of patients during the study.

A total of 100 patients undergoing mini-PCNL were enrolled and randomly assigned to the LP (LP, *n* = 50) or HP (HP, *n* = 50) groups (Fig. 1). Baseline demographic characteristics, comorbidities, and stone-related parameters were well-balanced between the groups. There were no significant differences in age, sex, body mass index, ASA classification, stone burden (size and volume), stone density, or skin-to-stone distance (Table 1).

**Table 1:** Patient demographics and preoperative stone characteristics.

Patient characteristics	Low-power group (*n* = 50)	High-power group (*n* = 50)
Age (years) (mean ± *SD*)	61.5 ± 12.5	61.5 ± 8.6
Gender: *n* (%)		
Male	26 (52.0)	24 (48.0)
Female	24 (48.0)	26 (52.0)
Laterality		
Left	28 (56.0)	27 (54.0)
Right	22 (44.0)	23 (46.0)
BMI (kg/m^2^) (mean ± *SD*)	25.5 ± 4.7	26.6 ± 4.4
History of previous stone intervention		
PCNL (*n*)	4	3
ESWL (*n*)	2	3
Diabetes mellitus: *n*	5	6
Hypertension: *n*	6	8
Dyslipidemia: *n*	6	5
ASA classification: *n* (%)		
Class 1	38 (76.0)	35 (70.0)
Class 2	10 (20.0)	12 (24.0)
Class 3	2 (4.0)	3 (6.0)
Stone volume (mm^3^) (mean ± *SD*)	2770.0 ± 1308.7	2888.0 ± 1232.0
Stone size (mm) (mean ± SD)	26.6 ± 7.5	27.2 ± 7.7
Number of stones, *n* (%)		
1	28 (56.0)	27 (54.0)
2	12 (24.0)	13 (26.0)
3	6 (12.0)	6 (12.0)
≥4	4 (8.0)	4 (8.0)
Skin-to-stone distance (mm) (mean ± SD)	83.8 ± 10.0	85.5 ± 15.9
Stone density (Hounsfield units) (mean ± SD)	998.0 ± 255.0	991.3 ± 318.2
Grade of hydronephrosis: *n* (%)		
No or mild	39 (78.0)	36 (72.0)
Moderate or severe	11 (22.0)	14 (28.0)
Number of accesses: *n* (%)		
Single	43 (86.0)	44 (88.0)
Multiple	7 (14.0)	6 (12.0)
Preoperative eGFR (mL/min/1.73 m²) (mean ± *SD*)	86.2 ± 18.2	87.5 ± 14.2

Note: Baseline characteristics are presented descriptively; no formal statistical comparisons were performed, in accordance with CONSORT recommendations.

Stone size refers to the maximum diameter of the largest stone measured on preoperative imaging.

**Abbreviations**: ASA, American Society of Anesthesiologists; BMI, body mass index; eGFR, estimated glomerular filtration rate; ESWL, extracorporeal shock wave lithotripsy; PCNL, percutaneous nephrolithotomy; SD, standard deviation.

Perioperative outcomes are summarized in Table 2. The HP group demonstrated significantly improved operative efficiency with a significantly shorter total operative time than the LP group (90.7 ± 18.7 vs. 112.1 ± 40.3 min; *P* = 0.001). Similarly, the laser activation time was significantly reduced in the HP group (1893 ± 499 vs. 2548 ± 1001 s; *P* = 0.001). Conversely, the total laser energy delivered was significantly higher in the HP group (37.6 ± 9.7 vs. 25.1 ± 9.8 kJ; *P* = 0.001).

**Table 2:** Perioperative outcomes and postoperative complications between the two groups.

Parameters	Low-power group (*n* = 50)	High-power group (*n* = 50)	*P*-value
Operative time (min) (mean ± *SD*)	112.1 ± 40.3	90.7 ± 18.7	0.001*
Fall in hematocrit (%) (mean ± *SD*)	3.3 ± 1.3	3.1 ± 1.2	0.504
Estimated blood loss (mL) (mean ± *SD*)	116.4 ± 89.3	126.0 ± 112.5	0.727
Blood transfusion requirement *n* (%)	3 (6.0%)	2 (4.0%)	0.646
Length of hospital stay (days) (mean ± *SD*)	3.5 ± 0.7	3.5 ± 0.8	0.902
Stone-free status (≤ 4 mm) *n* (%)	43 (86.0%)	44 (88.0%)	0.766
Zero-fragment status *n* (%)	33 (66.0%)	36 (72.0%)	0.518
Postoperative complications (Clavien–Dindo) *n* (%)			0.757
Grade I	11 (22.0%)	10 (20.0%)	
Grade II	5 (10.0%)	4 (8.0%)	
Total energy (kJ)	25.1 ± 9.8	37.6 ± 9.7	0.001*
Lasing time (sec)	2548.0 ± 1001.4	1893.0 ± 499.2	0.001*
J/mm^3^	10.9 ± 6.0	16.4 ± 10.2	0.002*
Ablation speed (mm^3^/s)	1.2 ± 0.6	1.6 ± 0.8	0.002*

Note: *Statistically significant.

Complications were classified according to the Clavien–Dindo classification.

**Abbreviations**: *SD*, standard deviation.

Regarding laser efficiency dynamics, the HP group achieved a significantly faster stone ablation speed (1.6 ± 0.8 vs. 1.2 ± 0.6 mm³/s; *P* = 0.002). However, when normalized to stone volume, the LP group was more energy efficient, requiring less energy per cubic millimeter of stone (10.9 ± 6.0 vs. 16.4 ± 10.2 J/mm³; *P* = 0.002).

Postoperative clinical outcomes were comparable between the two groups. The stone-free rate at 6 weeks showed no significant difference (LP 86.0% vs. HP 88.0%; *P* = 0.766). In addition, the zero-fragment rate was 66.0% in the low-power group and 72.0% in the high-power group, with no statistically significant difference between groups (*P* = 0.518). Safety profiles were similar, with no significant differences observed in estimated blood loss, hemoglobin drop, transfusion requirements, or length of hospital stay. The overall complication rate did not differ significantly (*P* = 0.757), with the majority being minor (Clavien–Dindo grade I–II) and distributed evenly between the groups. No Clavien–Dindo grade ≥ III complications were observed in either group.

## Discussion

This randomized controlled trial, conducted in a suction-assisted mini-PCNL setting where active suction was applied in both groups, demonstrated that the high-power Ho: YAG laser strategy, as applied in this study, was associated with significantly improved operative efficiency compared to low-power strategies. The observed 19% reduction in operative time and 26% reduction in laser activation time suggest that differences in laser settings, particularly higher-frequency pulse modulation, may contribute to more efficient apparent stone fragmentation under comparable operative conditions. Similar findings have been reported in recent studies comparing lithotripsy modalities, where higher-frequency pulse modulation or novel energy delivery systems achieved faster apparent ablation rates and improved intraoperative efficiency^9,11^.

Operative efficiency in mini-PCNL is multifactorial, dependent not only on fragmentation speed but also on fragment evacuation and visual clarity. The improved efficiency observed in the HP group in our study may be attributed to the synergy between high-frequency “pop-dusting” and suction-assisted evacuation. In contrast, the pop-corning technique used in the LP group tends to generate larger fragments within a confined space, which may prolong fragmentation and evacuation time. Recent multicenter evidence supports the role of suction in maintaining low intrarenal pressure and temperature, which are known to influence visibility and procedural safety^8,13^. Active suction systems have been reported to facilitate debris evacuation and may function as dynamic cooling systems, potentially mitigating risks associated with high-energy laser use. However, these mechanistic explanations remain inferential, as intrarenal temperature and pressure were not directly measured in this study.

Our findings highlight an “efficiency paradox” in suction-assisted mini-PCNL. The HP strategy achieved significantly greater time efficiency, with a shorter operative duration (mean 90.7 vs. 112.1 min, *P* = 0.001) and laser activation time (1893 vs. 2548 s, *P* = 0.001), yet required higher total energy delivery (37.6 vs. 25.1 kJ, *P* = 0.001). Consequently, although the apparent ablation speed was higher (1.6 vs. 1.2 mm³/s, *P* = 0.002), the apparent energy efficiency was lower, as reflected by a higher mean energy density (16.4 vs. 10.9 J/mm³, *P* = 0.002). This inverse relationship between time and energy efficiency is consistent with prior studies suggesting that energy efficiency may decline at higher laser settings due to energy dissipation into the irrigant medium and increased retropulsion^16,17^. Clinically, this higher energy demand may represent a justifiable trade-off, as it is associated with shorter operative times and improved surgical throughput, which are particularly relevant in high-volume endourological settings.

In addition to efficiency metrics, the trade-off between operative time and energy utilization may have implications for cost-effectiveness. While HP laser strategies were associated with shorter operative times, potentially improving operating room efficiency and surgical throughput, they also required higher total energy delivery and may be associated with increased energy consumption and potentially higher equipment-related costs. Conversely, LP strategies demonstrated greater energy efficiency, which may translate into lower energy consumption during the procedure. However, a formal cost-effectiveness analysis was beyond the scope of the present study, and these considerations should be interpreted as exploratory. Future studies incorporating economic evaluations are warranted to better define the cost-benefit balance between these strategies.

Thermal injury and elevated IRP are recognized risks of HP laser lithotripsy. In the present study, no increase in observed clinical complications was identified despite higher total energy delivery (up to 37.6 kJ), findings that are consistent with previously published clinical observations of suction-assisted mini-PCNL^13,18^. Beyond debris evacuation, active suction has been suggested to provide continuous hydrodynamic flow, which may contribute to heat dissipation and stabilization of intrarenal pressure. This dynamic flow may also improve endoscopic visibility by reducing the “snowstorm effect” from dust particles, thereby facilitating more efficient lithotripsy^19–22^. In the present study, although intraoperative visibility was not formally quantified, no clinically significant vision-related delays or interruptions in operative flow were observed in the high-power group. Furthermore, suction-based systems have been reported to shorten operative times, lower infection rates, and improve irrigation efficiency by maintaining low-pressure, high-flow circulation within the collecting system^23,24^. Recent international consensus statements have also highlighted the potential role of pressure-regulated suction systems in reducing procedure-related complications during high-energy lithotripsy^8^. However, it should be emphasized that these mechanistic considerations are based on external evidence, as intrarenal temperature and pressure were not directly measured in the present study.

Stone-free rates in our study were comparably high (> 86%) across both groups, consistent with previous reports indicating that laser settings predominantly influence operative efficiency rather than final stone clearance outcomes^25,26^. In addition to conventional stone-free rates, we also evaluated zero-fragment outcomes to provide a more stringent assessment of treatment efficacy. Although a numerically higher zero-fragment rate was observed in the high-power group, this difference did not reach statistical significance. This finding further supports that while laser strategy influences procedural efficiency, its impact on complete stone clearance may be limited when suction-assisted systems are employed. While emerging technologies such as the Thulium Fiber Laser (TFL) and Trilogy systems may further optimize fragmentation and reduce retropulsion, Ho: YAG laser systems—particularly when combined with pulse modulation and suction-assisted platforms—continue to provide a balanced approach in terms of efficiency, versatility, and clinical applicability^27^.

This study has several limitations. First, all procedures were performed by a single experienced surgeon. Although this strengthened technical consistency and reduced inter-operator variability, it may limit the generalizability of the findings to surgeons with different levels of experience, particularly those within the learning curve. In addition, a single-operator design introduces the possibility of performance bias, as operative efficiency and laser utilization may partly reflect individual technique, familiarity, or preference for specific laser settings. This is particularly relevant because operative time was the primary endpoint.

Second, while we hypothesized that suction mitigates thermal and pressure-related risks, intrarenal temperature and pressure were not directly measured in this study. Therefore, the mechanistic interpretation of these effects remains inferential.

Third, operative time was used as a surrogate marker of procedural efficiency. Although widely adopted, this parameter does not fully capture other relevant aspects such as surgeon ergonomics or fatigue, which may also influence procedural performance.

Fourth, the calculation of stone volume using two-dimensional formulas may introduce measurement variability, although randomization likely minimized inter-group bias.

Importantly, there was partial overlap in calculated laser power (wattage) between the LP and HP groups due to the combination of pulse energy and frequency settings. This limits the ability to interpret the findings as a pure comparison of laser power. Therefore, the results should be interpreted as a comparison of clinically applied laser strategies rather than isolated power effects.

In addition, laser efficiency parameters were calculated using preoperative stone volume, which assumes complete laser-mediated ablation of the stone burden. In suction-assisted mini-PCNL, however, a proportion of stone material may be removed through mechanical fragmentation and evacuation rather than laser ablation. Therefore, these metrics may overestimate true laser efficiency, particularly in cases with residual fragments or intact fragment evacuation. As such, these parameters should be interpreted as procedure-level efficiency metrics rather than direct measures of laser–stone interaction.

Furthermore, stone composition was not routinely analyzed, as it was not included in the predefined study protocol. Given that stone composition may influence fragmentation characteristics and laser efficiency, the absence of compositional data limits further interpretation of energy-related outcomes. However, due to the randomized design, this factor was likely balanced between groups.

Finally, stone-free status was not assessed in the immediate postoperative period. Although CT at follow-up provides a more accurate and sensitive evaluation of residual fragments, the absence of early imaging may limit assessment of immediate stone clearance dynamics.

## Conclusion

The high-power Ho: YAG laser strategy was associated with shorter operative and lasing times compared to low-power strategies within a suction-assisted mini-PCNL setting where active suction was applied in both groups, without an increased rate of observed clinical complications. These findings suggest that differences in laser settings, particularly high-frequency strategies, may improve procedural efficiency while maintaining a favorable perioperative safety profile within the limits of this study. However, these findings should be interpreted within the context of overlapping power settings and reflect differences in clinically applied laser strategies rather than isolated power effects.

## Electronic Supplementary Material

Below is the link to the electronic supplementary material.

Supplementary Material 1

Supplementary Material 2

## Data Availability

The datasets generated and/or analyzed during the current study are available from the corresponding author upon reasonable request.
