# Supplementary material for: Low- versus high-power holmium: YAG laser strategies affect operative efficiency in suction-assisted mini-PCNL in a randomized controlled trial
Source: Sci Rep. 2026 May 11;16:21440. doi: 10.1038/s41598-026-52978-7 (PMC13350882; doi:10.1038/s41598-026-52978-7)
Supplement: Supplementary file 2 — Supplementary Material 2 [file 41598_2026_52978_MOESM2_ESM.docx]

**Supplementary Table S1. Continuous perioperative outcomes presented as median (interquartile range)**

| **Variable** | **Low-power group (n = 50) Median (IQR)** | **High-power group (n = 50) Median (IQR)** |
| --- | --- | --- |
| Operative time (min) | 105 (85–135) | 88 (75–100) |
| Estimated blood loss (mL) | 90 (60–140) | 95 (65–150) |
| Total energy delivered (kJ) | 23 (18–30) | 36 (30–44) |
| Lasing time (sec) | 2400 (1900–3000) | 1800 (1500–2100) |
| Energy per volume (J/mm³) | 9.5 (6.5–13.5) | 15.0 (10.5–21.0) |
| Ablation speed (mm³/s) | 1.1 (0.8–1.5) | 1.5 (1.1–2.0) |
